# Surface Cement Concrete with Reclaimed Asphalt

**DOI:** 10.3390/ma16072791

**Published:** 2023-03-31

**Authors:** Małgorzata Linek, Magdalena Bacharz, Patrycja Piotrowska

**Affiliations:** Faculty of Civil Engineering and Architecture, Kielce University of Technology, Tysiąclecia Państwa Polskiego 7, 25-314 Kielce, Poland

**Keywords:** reclaimed asphalt pavement, airport pavements, cement concrete pavements

## Abstract

This research concerns the possibility of using reclaimed asphalt pavement as a substitute for conventional aggregate in cement concrete mixtures for roads and airfield applications. The advantages of using reclaimed asphalt pavement as a replacement for natural aggregates are presented. Economic and environmental aspects are indicated, including the reduction in the consumption of natural non-renewable sources of mineral aggregates, as well as reduction in transport costs and emissions of harmful greenhouse gases. The consistency of this recycled material with the idea of sustainable development in the construction industry is emphasized. The test results of the used reclaimed asphalt and the assessment of the effect of its amount on the change in mechanical, physical and strength parameters of cement concrete are presented. It has been shown that the addition of reclaimed concrete reduces selected parameters of cement concrete, but it is possible to use it in structures with less traffic load, taking into account the sustainable development policy.

## 1. Introduction

Technological development is closely related to and dependent on transport possibilities, especially in terms of the economics of given projects. Transport safety is the most important element that should be ensured at the stage of management of highway infrastructure (e.g., by using modern techniques [1,2,3]) but also at the stage of implementation and planning of the investment itself. The durability of communication, road and airport surfaces can be ensured at the stages of investment planning, structure design, incorporation of concrete mix and its proper care and maintenance during use. Many works [4,5,6] indicate the need to take into account these factors in terms of various technological processes and material solutions. In terms of materials, issues related to the current use of mineral aggregates are also indicated. Road and airport pavements differ significantly in terms of traffic load and the method of generated static, dynamic and thermal loads. The durability criterion of the concrete pavement is equated with the parameters of the concrete mix materials. One of the most important factors is the type and properties of the aggregate used in the crumb pile. In the works [7,8,9], attention was paid to the reactivity of the aggregates used, their influence on the parameters of cement concrete and the occurrence of damage. So far, natural aggregates are supplemented or replaced with alternative aggregates. In Poland, in accordance with the guidelines [10], granite grit should be used as the basic type of aggregate for mixtures intended for airport pavements. National requirements should be met in the case of road surfaces [11,12,13].

The results presented in [14] indicated that the difference in compressive strength results of analyzed aggregates (quartzite, granite, limestone and marble) was not significant for normal concretes, but for concretes with high durability the influence was significant. The authors of article [15] showed that the strength growth pattern of concrete with phyllite was similar to that of concrete with granite; however, this concrete obtained about 20% lower strength result. The paper [16] presents the possibility of using porphyry and amphibolite aggregate as a replacement for coarse aggregate for airport pavements. On the one hand, the analysis of the research described in [17] showed that concrete based on limestone and basalt aggregate is characterized by higher strength than concrete with gravel aggregate. On the other hand, the authors of the paper [18], examining the influence of the aggregate type, obtained the highest results for concrete with quartzite, gravel and granite, respectively. The paper [19] shows the influence of the shape of dolomite aggregate and the manner of its arrangement in the mixture on the strength of concrete.

It is estimated that in Europe the average production and consumption of natural aggregates is about 6.5 tons per capita [20]. Such a demand is related to the search for alternatives to non-renewable aggregate and possibilities of construction waste management. Currently, the processing of concrete waste into various types of aggregate is increasingly analyzed.

In the article authors consider the possibility of reusing the aggregate from recycled pavement. The use of recycled materials in transportation infrastructure corresponds with the sustainability concept due to its environmental and economic benefits.

## 2. Review of the Literature on the Topic

Reclaimed asphalt pavement (RAP) is the material created in the process of demolition or milling of a distressed flexible pavement which is an integral part in pavement renovation works. Asphalt destruct in its composition contains about 88% mineral aggregate, 7% filler and about 5% asphalt binder, thus it gives opportunities to reuse such amounts of mineral material [21]. The authors of the publication by Sigh et al., 2017 [22] indicate that the method of obtaining reclaimed asphalt pavement affects its properties, especially gradation. Many studies [23,24,25] reported that coarse reclaimed asphalt pavement is finer than natural coarse aggregates, on the other hand fine reclaimed asphalt pavement has been reported to be coarser than natural fine aggregates. Although pavement is commonly used in bituminous mixtures, it is worth checking the possibility of its use as a substitute for conventional aggregates in concrete mixes. It could provide many benefits such as: reduction in natural aggregate demands, reduction in greenhouse gas emissions, and savings in transportation costs [26].

The beginnings of work on the possibilities of using reclaimed asphalt pavement in cement concrete mixes date back to the 1990s. Delwar et al., 1997 [27] investigated the potential use of reclaimed asphalt pavement as an aggregate in concrete with Portland cement. Obtained compressive strength test results indicated that as the content of reclaimed asphalt pavement increases the compressive strength decreases, but concrete samples with reclaimed asphalt pavement were characterized by good shatter resistance properties.

In laboratory research Hassan et al., 2000 [28] analyzed the effect of reclaimed asphalt pavement on the characteristics of cement concrete using various compositions of mineral mixture in their laboratory batches. Authors took into account in the composition different proportions of reclaimed asphalt pavement with fine and coarse grain size. Studies have shown that the use of only coarse-grained reclaimed asphalt pavement in the mixture allows for better mechanical properties than in the case of replacing fine fractions with it. Huang et al., 2005 [29] discussed the effect of reclaimed asphalt pavement content in cement concrete mixtures on the stiffness and brittle cracking behavior of concrete composite. The thesis was made that the thin asphalt coat covering the aggregate in reclaimed asphalt material protected the aggregate grains by dissipating energy thus inhibiting the propagation of the crack and making the concrete with the reclaimed asphalt pavement more durable. In addition, Huang et al., 2006 [30] investigated the impact of a water-reducing admixture on concrete properties. They found that the use of an admixture had a positive effect on the mechanical properties of concrete with reclaimed asphalt pavement. The author of the work [31] presented the results of tests on fresh and hardened concrete with the addition of recycled aggregate. He pointed out that this aggregate is characterized by a lower density and higher water absorption than ordinary aggregate, and that it affects the parameters of concrete. This influence concerns lower compressive and bending strength, and lower value of the modulus of elasticity. As the amount of recycled aggregate increases, the shrinkage of concrete, water permeability and water absorption increase. Silva et al., 2019 [32] drew attention to the influence of the scale of the experiment on the obtained results of research on RA concrete intended for use on communication pavements. The authors of the work [33] presented the possibility of using recycled aggregate and fly ash. The obtained results show that the use of various contents of these components (up to 15% of fly ash and up to 10% of coarse aggregate from recycling) or their combination (up to 5% of total fly ash and recycled aggregate) allows obtaining concrete with parameters comparable to concrete without aggregates from recycling.

The research of [24] concerned the basic determinations of the mechanical properties of concrete, such as compressive strength, but also the influence of the destruct on the stiffness modulus, thermal expansion coefficient and shrinkage. The obtained results allowed the design of a computational model of a concrete slab. Numerical analysis in the FEACONS IV program, which was developed at the University of Florida, showed that concrete with the addition of reclaimed asphalt compared to the reference concrete has a lower ratio of stress to bending strength; therefore, the use of the additive has a positive effect on the performance of the analyzed material.

The use of reclaimed asphalt in concrete mixes is a promising material solution; therefore, the global interest in developing this technology is visible. The publications on this subject that have been published in recent years focus not only on the strength properties of the concrete, but more and more new aspects are analyzed. Brand and Al-Quadi [23] focused their research on the stiffness modulus of concrete with the addition of reclaimed asphalt. Issues related to the stiffness modulus were also discussed by [34]. The research by Abraham and Ransinchung [35] analyzed the effect of the addition of reclaimed asphalt on the properties of the cement matrix, including the structure of its air pores by mercury porosimetry.

The use of reclaimed asphalt as a substitute for natural aggregate brings many benefits, not only in terms of the natural environment, but also economic aspects [26]:

- Reducing the consumption of natural aggregates—destruct can be used both as a replacement for fine and coarse aggregate, and replacing natural aggregate with it in the amount of up to 50% does not cause a significant decrease in concrete properties [22,25,36].
- Reduction in greenhouse gas emissions—it is estimated that the production of 1 ton of aggregate generates about 1% of greenhouse gases [37].
- Conducting a sustainable concrete production process [38]. Sustainable development is by definition a resource-rational management that takes into account the needs of future generations.
- Savings in transport costs [22,36]—this is a huge savings, especially in situations when the natural aggregate bed is located at a considerable distance from the construction site and the concrete mixing plant.
- Savings in production costs—the amount of expenditure related to transport, extraction and preparation of natural aggregate significantly affects the cost of concrete production, it is estimated that it is possible to reduce the production cost of 1 m^3^ of concrete by up to 45% by using reclaimed asphalt as a substitute for natural aggregate in the concrete mix [25].

Despite the indicated advantages of using an alternative material in the form of reclaimed asphalt, the authors of the related publications indicate the lack of clear guidelines as to the possibility of using this additive in concrete mixes. An additional problem is the lack of awareness and concerns of contractors and investors regarding the quality and durability of objects made of cement concrete with reclaimed asphalt. However, the growing interest of scientists and the amount and scope of research work on the properties and possibilities of using reclaimed asphalt in cement concrete may, however, increase the interest in this technology [26].

In order to systematize the knowledge gained so far in the experimental field, selected parameters of the concrete mix and the concrete composite were compiled, which were the results of the analyses of researchers dealing with the issue of using reclaimed asphalt in concrete mixes.

Recent publications also focus on possible applications of reclaimed asphalt for rolled concrete (RCC-Roller-Compacted Concrete), research in this area was conducted by Debbarma, Ransinchung and Singh [39]. The possibility of using up to 50% of reclaimed asphalt for rolled concrete is recommended in the research by Settari, Debieb, Kadri and Boukendakdji [40].

Obtained results indicated that further research on use of reclaimed asphalt in rigid surfaces made of cement concrete is justified.

This research is aimed at checking the possibility of using reclaimed asphalt pavement for concrete mixtures in relation to polish road national standards.

## 3. Purpose and Scope

The aim of this research was to demonstrate the consistency between the internal structure of the concrete composite containing reclaimed asphalt and its mechanical parameters.

## 4. Durability Criteria for Road and Airport Pavement Concrete

Safe exploitation of concrete pavements is possible in terms of concrete that meets the functional, strength and durability requirements during the expected life cycle of the structure. In the context of road pavements, the static and dynamic loads generated by vehicles in conjunction with the stresses occurring in concrete slabs are included in the scope of structure design. In the airport systems, the forced thermal loads generated during the take-off stage of the aircraft are additionally taken into account. In terms of durability, these are multi-criteria issues that should be considered individually, as pointed out by the authors of [41,42,43,44,45].

## 5. Materials and Methods

### 5.1. The Scope of Laboratory Tests

Laboratory tests included determination of the impact of the use of reclaimed asphalt on the change in selected paving concrete parameters. The reclaimed material selected in the exploratory tests (discussed in [46]) was used. The composition of two concrete mixes for pavements was designed, taking into account the impact of the external environment in terms of exposure classes: XF4 (freeze/thaw attack with or without de-icing agents—high water saturation, with de-icing agent or sea water), XC4 (corrosion induced by carbonation—cyclic wet and dry), XA2 (chemical attack—moderately aggressive chemical environment). The design assumptions included a concrete class of C30/37 and a w/c ratio of up to 0.4. The basic parameters of concrete mixes, i.e., consistency class, air content and density of the mix, were determined. Selected physical, mechanical, performance and internal microstructure parameters were determined for the hardened concrete. The material requirements applicable in Poland for the composition of concrete intended for traffic pavements, with a distinction between road and airport structures, are summarized in Table 1.

### 5.2. Aggregates and Asphalt Destructs

Density and water absorption were determined for aggregates according to PN-EN 1097-6 [50] using the pycnometric method—Table 2. The aggregates used meet the requirements of PN-EN 13877-1 [11], PN-EN 12620 [56], and the requirements listed in the specification D-05.03.04 [57], for the upper and lower layers of the KR3-4 road surface. Due to the resistance to crushing by the Los Angeles drum method, according to PN-EN 1097-2 [48], the crushing resistance category for granite aggregate was LA30. These parameters also meet the requirements for airfield pavements according to the defense standard NO-17-A204 [10], where the minimum acceptable category of resistance to aggregate crushing is LA40. Coarse aggregate was assessed for alkaline reactivity according to the accelerated method PB/1/18 [58]. In this method, the assessment of aggregate reactivity was carried out on the basis of the average change in the length of the samples after 14 days of conditioning in 1 M NaOH solution at 80 °C. It was found that the aggregate did not show reactivity, and thus it was assigned the required R0 category because the change in the length of the sample was less than 0.10%.

The grain composition of the aggregates was determined in accordance with the requirements of PN-EN 12620 [56] and PN-EN 933-1 [47]. Aggregates meet the requirements for aggregates intended for the construction of road surfaces according to PN-EN 12,620 [56], PN-EN 206 [13], NO-17-A204 [10] and specification D-05.03.04 [57]. The graining category according to PN-EN 13,043 [54] for coarse granite aggregate is G_C_85/20 and for natural sand is G_F_85. The percentage share of individual grain sizes is summarized in Table 3.

Specific selected parameters of reclaimed asphalt and asphalt extracted from it are summarized in Table 4. The selection of the most advantageous type of asphalt destruct was discussed in article [46]. In the case of binder samples, the presence of a modifier was not found, as the determination of elastic recovery reached a value of 16%, and the presence of elastomers in the binder can be concluded when the tested feature is at least 50%.

For reclaimed asphalt, the grain composition and the maximum U size of the aggregate were determined. The reclaimed asphalt was classified as FM1/0.1 (the maximum U size was 22.4 mm and no foreign materials from groups 1 and 2). The grain size of the used reclaimed asphalt is shown in Table 5.

### 5.3. Components of the Concrete Mix

In the composition of the concrete mix, cement CEM I 42.5 was used as a binder, which meets the standard requirements for reduced alkalinity. The cement content was determined on the basis of the adopted design assumptions and standard requirements [10,11,13] in the amount of 370 kg/m^3^. The composition of the mix uses tap water that meets the requirements of [55], and admixtures that improve the parameters of the mix and hardened concrete. A plasticizing admixture with a density of 1.14 kg/dm^3^, pH of 5.5 and chloride ion content below 0.10% was used. An air-entraining admixture based on modified tensides and root resins was also used, in which the content of chloride ions was less than 0.10%. The compositions of concrete mixes are presented in Table 6. The designation of the reference concrete (without the addition of reclaimed asphalt) was adopted as C-C. On the other hand, the designation of concrete with the addition of reclaimed material in the amount of 20% was C-RA.

### 5.4. Research Methodology

The concretes were made in accordance with the requirements of a series of standards [10,13,59,60,61]. The care stage included conditioning the samples in standard conditions for a certain number of days (1, 7, 14 and 28) and then devoting them to destructive testing. The research cycle was divided into four stages. In the first stage, the physical parameters of concretes and the impact of reclaimed asphalt on their change were determined. The density of concrete was determined according to the standard PN-EN 12390-7 [62], water absorption according to [10] and the depth of water penetration under pressure according to PN-EN 12390-8 [63]. As part of the second stage, tests were carried out on the impact of the addition of reclaimed asphalt on mechanical parameters, i.e., compressive strength according to [64], flexural strength according to [65], tensile splitting strength [66] and modulus of elasticity according to [67]. In the third stage, the surface peel strength was determined according to [68] and the frost resistance of concrete according to [10] (by two methods: internal frost resistance after 200 cycles of freezing and thawing, and resistance to surface flaking after 56 cycles of freezing and thawing). In the fourth research stage, changes in the internal structure of the cement composite due to the presence of reclaimed asphalt in the composition of the mix were analyzed. The procedure for preparing samples for observation in the SEM scanning electron microscope and CT computed tomography was consistent with that described in [69,70].

The compared series of C-C and C-RA concretes were analyzed in accordance with the assumptions of the Student’s t-test, each time for at least six samples in one test series. It was assumed that the number of series is constant and the distribution of results in each of the analyzed groups is compared with the normal distribution. The basic statistical parameters, arithmetic mean, standard deviation and coefficient of variation were determined according to the procedure presented in [45].

In the research process, various types of samples were investigated, depending on their purpose and the test performed, which, together with the geometric characteristics, are summarized in Table 7.

## 6. Results and Discussion

### 6.1. Physical Parameters

It was found that the concrete with the addition of reclaimed asphalt of the C-RA series was characterized by a lower density compared to the control concrete (Table 8).

The depth of water penetration under pressure is one of the durability parameters of structural concrete in a road engineering structure. At the same time, a beneficial effect of reclaimed material on the reduction in water absorption under pressure and the depth of concrete penetration was found (Table 9). This is important in the case of the operation of surface structures in real environmental conditions and is a positive phenomenon.

### 6.2. Mechanical Parameters

**Figure 1:**
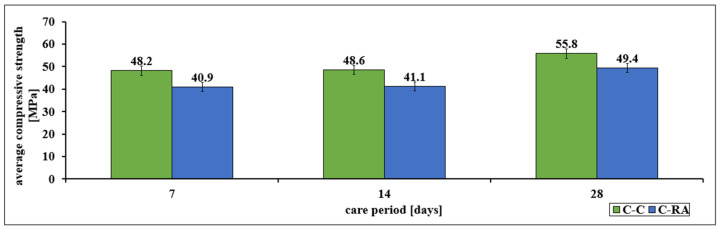
Change in the average compressive strength of concretes of the C-C and C-RA series as a function of the curing period.

In the case of mechanical parameters, a decrease in the examined features was found, regardless of the analyzed parameter. The decrease in compressive strength was found in all three test periods (after 7, 14 and 28 days). The highest decrease was recorded in the case of early ripening, after 7 and 14 days, which was about 15% (Figure 1). Analyses performed after 28 days of curing showed that the average compressive strength decreased by more than 11%. This decrease translated into a decrease in the concrete class from C40/50 for C-C series concrete to C35/45 for C-RA series concrete.

Statistical parameters characterizing individual series are presented in Table 10.

The results obtained from the compressive strength test were characterized by low values of the standard deviation, which means that the average values obtained in laboratory tests are statistically representative.

**Figure 2:**
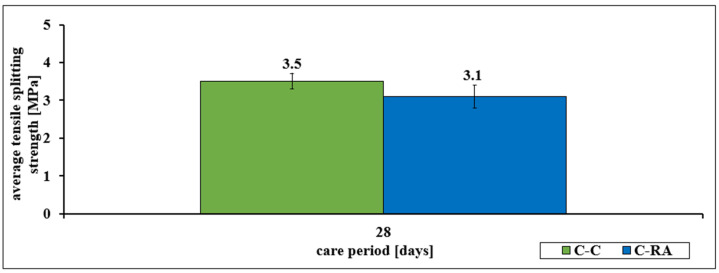
Change in the average tensile splitting strength of concretes of the C-C and C-RA series.

**Figure 3:**
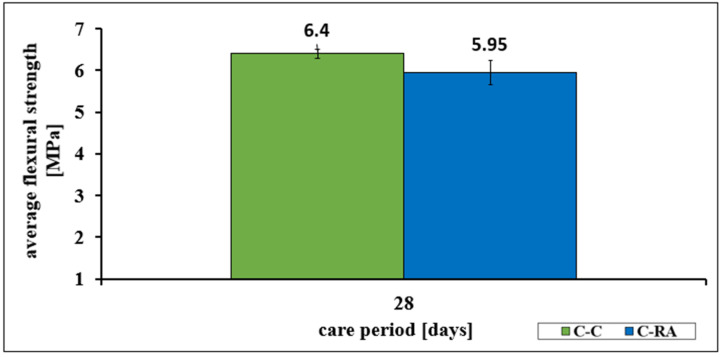
Change in the average flexural strength of concretes of the C-C and C-RA series.

Similar dependencies occurred in the assessment of average flexural strength and average tensile splitting strength of the C-RA series concrete in relation to the C-C series concrete. A decrease in strength of more than 11% in the first case and 7% in the second case was found (Figure 2 and Figure 3).

**Figure 4:**
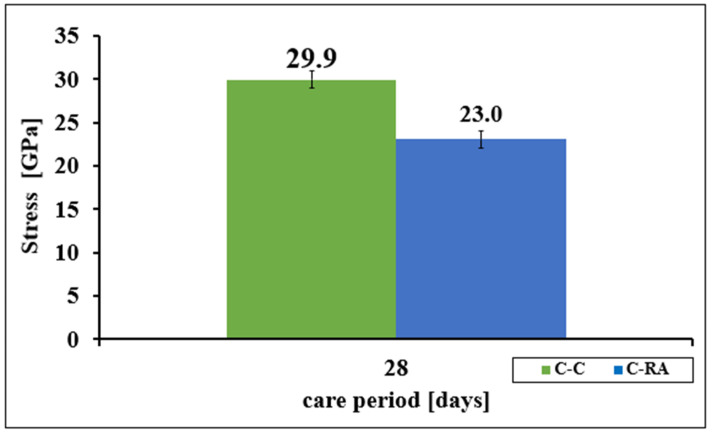
Change in the average Young’s modulus of concretes of the C-C and C-RA series.

Approximately 30% higher values of Young’s modulus results were measured in concrete C-C. The results were characterized by a slight differentiation, the coefficient of variation for concrete C-C was 1.3%, and 4.0% for concrete C-RA (Figure 4).

**Figure 5:**
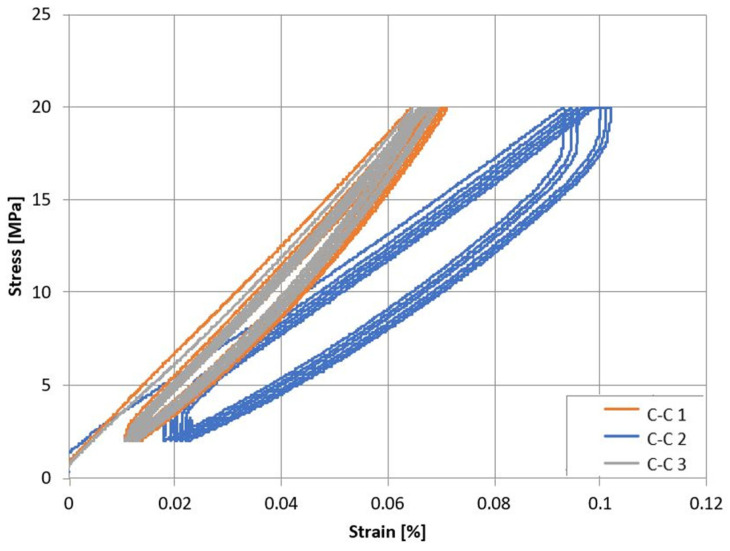
Stress–strain dependence for C-C series concrete.

**Figure 6:**
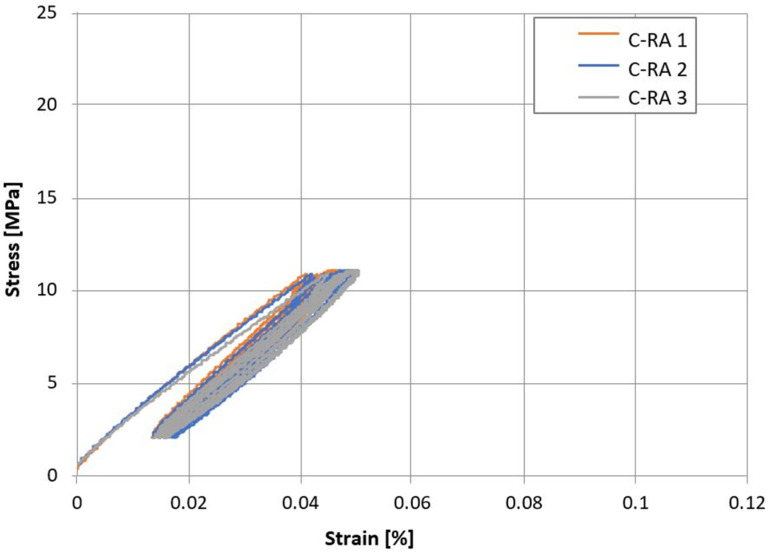
Stress–strain dependence for C-RA series concrete.

The stress–strain dependence for the analyzed concrete series C-C and C-RA is shown in Figure 5 and Figure 6. In the case of modified concrete, the obtained values are lower.

**Figure 7:**
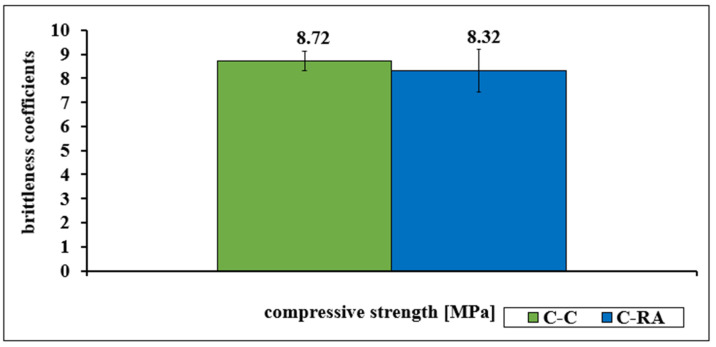
The dependencies of the brittleness coefficient for C-C and C-RA concrete.

The presented test results clearly indicate that the fracture toughness of C-RA concrete is higher than that of C-C concrete. The index for the C-C series concrete was 0.115, and for the C-RA concrete it was 0.120. These results are also consistent with the specified brittleness coefficients of the analyzed concretes, as shown in Figure 7. The presented relationship shows that the C-RA series concrete is more susceptible to shrinkage deformations. Higher resistance to deformation can result in a longer service life of the concrete in the structure. This is a particularly important feature in the case of demanding environmental conditions, in which temperature fluctuations oscillate around 0 degrees, because they can affect the appearance of additional stresses in concrete slabs.

### 6.3. Durability Parameters

The pull-off method is a measure of the concrete’s pull-off strength and is one of the methods for evaluating the durability of a structure. Based on the obtained results (Table 11), a lower resistance to peeling off the surface layer was found in the case of C-RA series concrete. It should be noted that two series of concrete met the minimum guidelines of the standard for values above 2.0 MPa.

The assessment of concrete resistance to freezing and thawing cycles showed that both concrete series meet the requirements for traffic surfaces (Table 12).

By analyzing the durability parameters, it was shown that the C-C series concrete obtained a higher durability factor than the C-RA series concrete.

**Figure 8:**
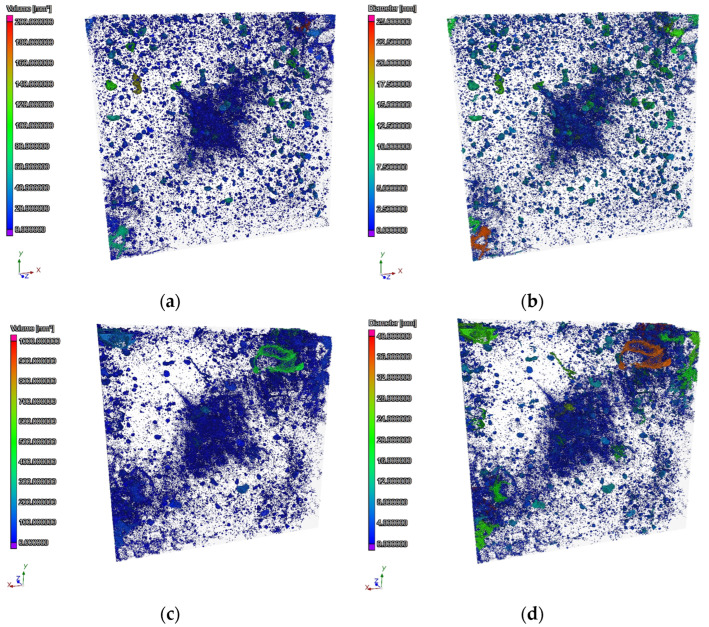
Examples of concrete cross-sections in terms of pore volume ((**a**) for C-C series concrete and (**c**) for C-RA series concrete) and in terms of pore diameter ((**b**) for C-C series concrete and (**d**) for concrete series C-RA).

The CT observation enabled a detailed assessment of changes in the total porosity content of C-C and C-RA series cement concrete. The obtained parameters are summarized in Table 13. Selected cross-sections of concrete of the C-C and C-RA series are shown in Figure 8.

Based on the analysis of the obtained results, it was found that the total content of air voids in the C-C series concrete is higher than in the C-RA concrete. However, the content of pores with diameters below 300 µm and round pores, which increase the level of frost resistance in cement concrete, is higher in the C-RA series concrete.

**Figure 9:**
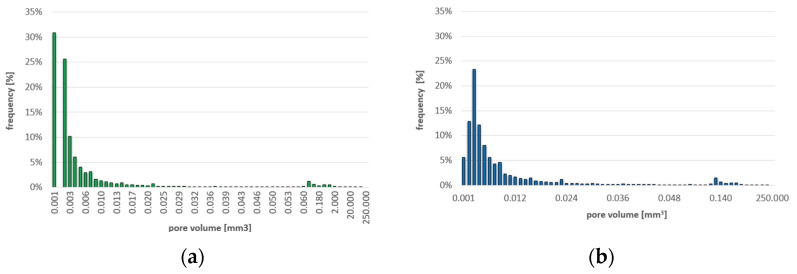
Frequency of pores with different volumes: (**a**) for C-C series concrete, (**b**) for C-RA series concrete.

**Figure 10:**
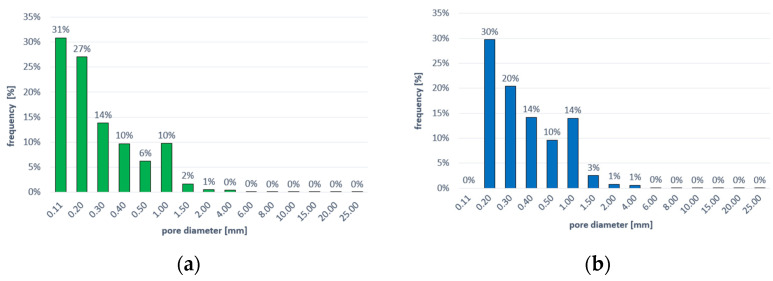
Frequency of pores with different diameters: (**a**) for C-C series concrete, (**b**) for C-RA series concrete.

**Figure 11:**
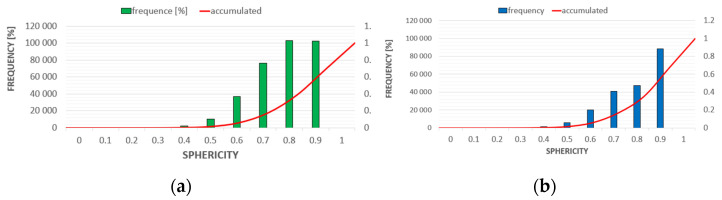
Frequency of pores with different sphericity ranges: (**a**) for C-C series concrete, (**b**) for C-RA series concrete.

The differences in the frequency of air pores of different volumes and diameters are shown in Figure 9 and Figure 10, respectively. The frequency of pores with different sphericity ranges is shown in Figure 11.

## 7. Conclusions

Based on the obtained results and their analysis, the following conclusions were formulated:

1. The use of reclaimed asphalt in the concrete mix allows for comparable air-entrainment parameters, density and consistency classes.

The air content determined by the pressure method for the C-C reference mixture was 4.6% and for the C-RA series mixture it was 4.8%. Both series of mixtures met the minimum content requirements ranging from 4.5% to 6%. The used reclaimed asphalt consisted of visible agglomerations of aggregate combined with an asphalt binder, which contributed to an increase in the air content in the C-RA mix.

The use of reclaimed asphalt reduces the density of the concrete mix (from 2389 kg/m^3^ for the C-C series to 2370 kg/m^3^ for the C-RA series). This is due to the fact that reclaimed asphalt has a lower density than the granite aggregate used in this experimental program.

2. The use of reclaimed material reduces the density of hardened concrete in all analyzed periods. Reducing the density of C-C series concrete from 2430 kg/m^3^ to 2407 kg/m^3^ for the C-RA series is related to the difference in the density of granite aggregate (2.65 kg/m^3^) and reclaimed asphalt (2.56 kg/m^3^).
3. In the case of physical parameters, a beneficial effect of the addition of reclaimed material was observed. Water absorption of the C-RA series concrete (3.9%) was lower than that of the control concrete (4.2%). A similar relationship occurred with regard to testing the depth of water penetration under pressure. The tested feature for the C-RA series was 12 mm and for the C-C series it was 23 mm.
4. In the case of mechanical parameters, a decrease in the tested concrete properties was observed due to the presence of reclaimed material.

The compressive strength of the C-RA series concrete was significantly lower than that of the C-C series control concrete in all analyzed research periods. In the case of the first 7 days, this decrease was about 15%, after 14 days it was also 15% and after 28 days it was 12%. The use of reclaimed material resulted in a reduction in the strength class from C40/50 for the C-C series to C35/45 for the C-RA series.

Similar relationships were also observed for the characteristics of bending strength (decrease of more than 7%) and tensile strength at splitting (decrease of more than 11%). For the latter feature, both series of concretes meet the requirements for road surface concretes with traffic categories from KR1 to KR4. However, the C-RA series does not meet the requirements for airport pavements.

5. In the case of durability features, no significant effect on the change in the tested concrete parameters due to the addition of reclaimed material was observed.

The frost resistance parameters of both concrete series are comparable and meet the requirements for road and airport pavements.

The lower values of the peel strength of the surface layer of the C-RA series concrete (2.35 MPa) in relation to the C-C series concrete (2.92 MPa) indicate a potentially lower resistance of the surface concrete. Especially in the case of more heavily loaded surfaces, such as airport pavements, this may shorten the service life of the structure. It should be noted, however, that in the case of both concrete series, the minimum required value of 2.0 MPa was achieved.

## Figures and Tables

**Table 1:** Material requirements for the composition of concrete intended for road and airport pavements—surface layer [45,46].

Parameters		Pavements	
		Roads	Airports
Coarse aggregate		According to:	According to:
	Dust content and dust quality	[47]	[47]
	Crushing resistance	[48]	[48]
	Polishing resistance	[49]	[49]
	Abrasion resistance	[50]	[50]
	Grain density and absorbability	[51]	[51]
	Frost resistance	[52]	[52]
	Chemical composition	[53]	[53]
Fine aggregate		According to [11]	According to [10]
	Grain-size distribution	Limit curves	Limit curves
	Cement-minimum class	32.5	32.5
	Type of cement	CEM I, CEM II According to [54]	CEM I According to [54]
	Water	According to [55]	According to [55]

**Table 2:** Aggregate parameters.

Parameters	Natural Sand 0/2	Granite Grits 2/8	Granite Grits 8/16
Density [Mg/m^3^]	2.63	2.65	2.65
Water absorption [%]	0.12	0.54	0.54
Resistance to fragmentation			LA_30_
Alkaline reactivity			R0

**Table 3:** Grain composition of aggregates.

# [mm]	Fine Aggregate 0/2	Granite Grits 2/8	Granite Grits 8/16
31.5	-	-	-
22.4	-	-	-
16.0	-	-	9.4
11.2	-	-	46.1
8.0	-	6.6	34.6
5.6	-	42.6	7.9
4.0	0.4	22.1	1.3
2.0	4.1	25.2	0.6
1.0	10.5	2.7	0.1
0.5	28.2	0.4	0.0
0.25	46.5	0.2	0.0
0.125	9.6	0.1	0.0
0.063	0.7	0.1	0.0
Σ	100	100	100

**Table 4:** Parameters of reclaimed asphalt.

Parameters	Reclaimed Asphalt
Density [Mg/m^3^]	2.556
Binder content [%]	4.5
Parameters	Extracted Asphalt
Penetration at 25 °C [0.1 mm]	45
Softening point [°C]	58.9
Elastic recovery [%]	16

**Table 5:** Graining of reclaimed asphalt.

Sieve dimension # [mm]	22.4	16.0	8.0	2.0	1.0	0.063
Reclaimed asphalt [%]	100	89	63	37	19	9

**Table 6:** Mixture composition.

Components [kg/m^3^]		Concrete C-C	Concrete C-RA
Cement		370	370
Water		133	133
Fine aggregate		553	549
Coarse aggregate	2/8 mm	830	628
	8/16 mm	590	390
Reclaimed asphalt		0	392 *
Air-entraining admixture		1.7	1.7
Plasticizing admixture		2.6	2.6

* Percentage share of reclaimed content, taking into account changes in the density of aggregates and the share of individual grain size fractions.

**Table 7:** List of parameters of hardened concrete samples intended for testing.

Tested Parameters	Type of Experimental Study	Research Period [Days]	Sample Type	Sample Dimensions [mm]
Physical parameters	Density	14; 28	Cubic samples	150 × 150 × 150
	Depth of penetration	28	Cubic samples	150 × 150 × 150
	Water absorption	28	Cubic samples	150 × 150 × 150
Mechanical parameter	Compressive strength	7; 14; 28	Cubic samples Cylindrical samples	150 × 150 × 150 150 × 300
	Tensile splitting strength	28	Cylindrical samples	150 × 300
	Flexural strength	28	Beam samples	150 × 150 × 700
	Stress—Young’s modulus	28	Cylindrical samples	150 × 300
Durability parameters	Pull-off	28	Rectangular samples	300 × 300 × 100
	Frost resistance	28 + 200 cycles *	Cubic samples	100 × 100 × 100
		28 + 56 cycles **	Rectangular samples	150 × 150 × 50
Microstructure parameters	SEM	28	Fractures of samples	10 × 10 × 10
	TC	28	Cubic samples	100 × 100 × 100

* The test consists of 200 successive cycles of freezing (in air at −18 ± 2 °C) and thawing (in water at +18 ± 2 °C). ** The test involves consecutive 56 freeze–thaw cycles in water.

**Table 8:** Density of hardened concrete results.

Parameters	Concrete	
	C-C	C-RA
Density of hardened concrete after 14 days [kg/m^3^]	2420	2400
Density of hardened concrete after 28 days [kg/m^3^]	2430	2410

**Table 9:** Depth of penetration of water results.

Parameters	Concrete	
	C-C	C-RA
Depth of penetration of water [mm]	23	12
Water absorption [%]	4.2	3.9

**Table 10:** Statistical parameters for the compressive strength feature.

Statistical Parameters	Concrete					
	C-C			C-RA		
	7	14	28	7	14	28
Minimal value [MPa]	45.5	45.1	52.5	37.8	39.3	45.9
Maximum value [MPa]	51.3	51.8	58.7	43.9	43.2	51.6
Standard deviation [MPa]	2.37	2.30	2.25	2.19	1.50	2.44

**Table 11:** Change in the average pull-off strength of concretes of the C-C and C-RA series.

Parameters	Concrete	
	C-C	C-RA
Pull-off strength [MPa]	2.92	2.35

**Table 12:** Resistance of C-C and C-RA series of concretes to frost resistance cycles.

Parameters	Test Method	Allowable Maximum Value Specified in the Standard	Concrete	
			C-C	C-RA
Strength decrease [%]	Internal frost resistance	20	4.96	3.33
Weight loss [%]		5	0.07	0.70
Weight loss [kg/m^2^]	Surface flaking resistance	0.01	0.00	0.003

**Table 13:** Porosity parameters for C-C and C-RA series concrete, the volume of the samples was 1687.5 cm^2^.

Parameters	j.m.	Concrete	
		C-C	C-RA
The content of voids in the entire sample	%	1.0584	1.0186
Pore content with a diameter of less than 300 μm	%	0.0326	0.0439
Round pore content (sphericity ranging from 0.8 to 1.0)	%	0.0053	0.0076

## Data Availability

Not applicable.
